# Optimized Performance Parameters for Nighttime Multispectral Satellite Imagery to Analyze Lightings in Urban Areas

**DOI:** 10.3390/s20113313

**Published:** 2020-06-10

**Authors:** Jasper de Meester, Tobias Storch

**Affiliations:** German Aerospace Center (DLR), Earth Observation Center (EOC), Münchener Str. 20, 82234 Weßling, Germany; demeesterjasper@hotmail.com

**Keywords:** nighttime remote sensing, satellite image simulation, urban area, multispectral band optimization, high spatial resolution, lighting parameter, lighting type classification

## Abstract

Contrary to its daytime counterpart, nighttime visible and near infrared (VIS/NIR) satellite imagery is limited in both spectral and spatial resolution. Nevertheless, the relevance of such systems is unquestioned with applications to, e.g., examine urban areas, derive light pollution, and estimate energy consumption. To determine optimal spectral bands together with required radiometric and spatial resolution, at-sensor radiances are simulated based on combinations of lamp spectra with typical luminances according to lighting standards, surface reflectances, and radiative transfers for the consideration of atmospheric effects. Various band combinations are evaluated for their ability to differentiate between lighting types and to estimate the important lighting parameters: efficacy to produce visible light, percentage of emissions attributable to the blue part of the spectrum, and assessment of the perceived color of radiation sources. The selected bands are located in the green, blue, yellow-orange, near infrared, and red parts of the spectrum and include one panchromatic band. However, these nighttime bands tailored to artificial light emissions differ significantly from the typical daytime bands focusing on surface reflectances. Compared to existing or proposed nighttime or daytime satellites, the recommended characteristics improve, e.g., classification of lighting types by >10%. The simulations illustrate the feasible improvements in nocturnal VIS/NIR remote sensing which will lead to advanced applications.

## 1. Introduction

Nocturnal optical remote sensing in the visible and near infrared (VIS/NIR) of the electromagnetic (EM) spectrum is largely inferior both to its daytime counterpart as well as to nighttime remote sensing in the thermal infrared. Even if there is a large gap in terms of the amount and the diversity of available missions and products, there exists demand for such nighttime products. The interest in such products is growing as evident from the increasing number of applications [1]. These include the monitoring of human settlements and urban dynamics, the estimation of demographic and socio-economic information, light pollution and its influence on ecosystems and human health and astronomical observations, energy consumption and demands, detection of gas flares and forest fires, natural disaster assessment, and the evaluation of political crises and wars [2]. Most of these applications are derived from data linked to artificial lights which emit mainly in the VIS/NIR. A stronger focus on optical nighttime remote sensing is, therefore, well-founded. However, the aim of the first satellite sensor imaging low-light data, namely DMSP-OLS in 1976 [3], was to collect global cloud cover data day and night, detecting nocturnal VIS/NIR emission sources was a widely used by-product. For example, to derive essential socio-economic information, the lighting type is, however, a much stronger indicator of economic growth than solely the intensity of light as used in most studies [4]. In 2011, a considerable improvement in spatial resolution from 2700 to 750 m and detection limits from 5 × 10${}^{-9}$ to 2 × 10${}^{-10}\;$W m${}^{-2}$sr${}^{-1}$ nm${}^{-1}$ was possible with the arrival of its follow-on, the NPP-VIIRS-DNB yet with a daily global coverage [5]. In addition to these panchromatic (500–900nm, [5], for NPP) space-based nighttime images, trichromatic ones come in the form of photographs with a spatial resolution between 10 and 200 m taken by astronauts aboard the ISS irregularly since 2003 [6]. Other panchromatic data are acquired only over China frequently with a spatial resolution of 130 m by LJ1-01 since 2018 [7] and of 0.7 m by EROS-B sporadically since 2013 [8]. Other data with multiple spectral bands are sporadically acquired only with a spatial resolution of 120 m by AC-5 (AC-4 with similar spectral resolution) since 2013 [9] and of 0.9 m by JL1-3B (JL1-07/08 with similar spectral resolution) since 2017 [10]. Furthermore, sporadically acquired nighttime images of operational daytime missions reveal detection limits, e.g., for Landsat-8, of above $4\times 10^{-4}\;$W m${}^{-2}$sr${}^{-1}$nm${}^{-1}$ only for the multiple spectral bands [11].

The need for finer spectral and spatial resolutions was expressed many times. For example, a high-pressure sodium (HPS) lamp is indistinguishable from a light emitting diode (LED) lamp in panchromatic images and a conversion from HPS to LED is even incorrectly observed as a decrease in radiant flux for typical panchromatic images ([12], for Milan, Italy). For example, a street with one lamp every 25 m is indistinguishable from a street with two lamps every 50 m in 100 m resolution according to the Nyquist sampling theorem. Despite a proposal for a Nightsat mission in 2007 [13], however, there is still no space-based nighttime VIS/NIR mission up with spatial resolution less than 100 m, multiple spectral bands, and a global coverage.

For daytime imaging, for example, with four spectral bands typically blue (457–523 nm), green (542–578 nm), red (650–680 nm), and NIR1 (784–900 nm) are recommended ([14], for Sentinel-2) and a panchromatic band (450–800 nm). Furthermore, spectral bands red edge (705–740 nm) and NIR2 (960–1040 nm) are suggested. For nighttime imaging typically the same sensors are used. In order to determine optimal spectral and radiometric characteristics for dedicated nighttime VIS/NIR imaging, it is important to note that available data, for example based on airborne campaigns, provide only panchromatic imagery with high radiometric resolution ([15], for Berlin, Germany) or high spectral imagery with only low radiometric resolution ([16], for Las Vegas, NV, USA). Hence, these sources do not satisfactorily determine the optimal sensor parameters and performances; instead, an end-to-end sensor simulation is required with controlled environments to perform a realistic and precise examination.

The objective of this article is to recommend spectral and radiometric nighttime sensor parameters that are needed to support the community’s requirements, as well as those of the lighting engineering community and the general public, with a main focus on urban environments and the detection and differentiation of artificial outdoor irradiance sources at necessarily high spatial resolution.

**Figure 1:**
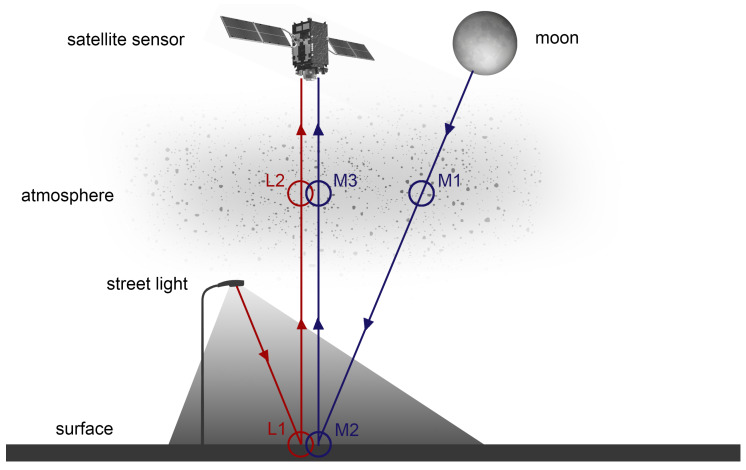
Model of propagation of nighttime radiation in optical remote sensing (under cloud-free conditions) with: (M1) downward atmosphere interaction with moon electromagnetic (EM) waves; (M2) surface interaction with moon EM waves; (M3) upward atmosphere interaction with moon EM waves; (L1) surface interaction with lamp EM waves; (L2) upward atmosphere interaction with lamp EM waves.

Therefore, Section 2 analyzes the elements affecting the sensor signal, namely the natural (e.g., moon) and artificial (e.g., street light) nighttime radiation sources, their interactions with the surface (M2 and L1) and atmosphere (M1, M3, and L2), and the satellite sensor itself as illustrated in Figure 1. For daytime similar considerations were widely investigated, e.g., by [14]. For nighttime similar considerations were rarely investigated, e.g., by [17] based on their findings on spectrometer measurements of outdoor lighting spectra. However, as only light source spectra were taken into account, a large part of the complexity is ignored by neglecting, for example, the variability in surface reflectances, atmospheric composition and sensor noise. For instance, two identical HPS or LED will look different when illuminating a patch of grass compared to a stretch of asphalt. Similarly, they will look different under hazy conditions compared to a clear night. Additionally, the number of spectral band combinations that the authors have considered was limited to eight and does not cover the full range of possibilities. Nevertheless, their recommendations are a reference for the considerations. Here, radiances are constructed which combines spectra from different lighting types, different intensities, different surface types, and different atmospheric compositions. In other words, it generates theoretical reference top-of-atmosphere (TOA) radiances for different conditions to know which signals arrive at a space-based sensor at night.

Section 3 utilizes this data to answer the question, if it is possible to discriminate between different radiation sources from space-based images and at what spectral and radiometric resolutions. The complexity exceeds that of the traditional classification task, where the illumination source is known (e.g., sunlight) and the surface object types are unknown. In the nighttime case, the illumination source is also unknown and furthermore, irradiances produced by artificial lights are sometimes mixed with moonlight. It is, therefore, important to know how different moon characteristics affect TOA radiances and the discrimination of lighting types. Knowing the type of radiation source sheds light on a number of important light characteristics. Some essential considerations of lighting, however, are not linked to lighting type on a one-to-one basis. As a consequence, it is necessary to consider the dominant criteria in the planning of nighttime lighting, and how far these lighting quality indices, namely luminous efficacy of radiation (with radiant flux and luminous flux), spectral G index, and correlated color temperature, are derivable [18]. Hence, the results of the simulations performed for various spectral and radiometric parameters and performances are analyzed, namely optimal spectral bands are derived (and typical TOA radiances are considered for the optimal radiometric resolution). While the radiances focus on homogeneous single-pixel environments, this approach does not, however, take into account any spatial information such as a lamp’s intensity distribution pattern or the overlapping of different lights. Therefore, also the spatial resolution is discussed.

Finally, Section 4 concludes the findings and recommendations for sensor parameters as well as the overview for future research.

## 2. Materials and Methods

### 2.1. Natural Radiation Sources

There are in fact a number of natural light sources emitting light in the VIS/NIR of the EM spectrum during nighttime.

**Figure 2:**
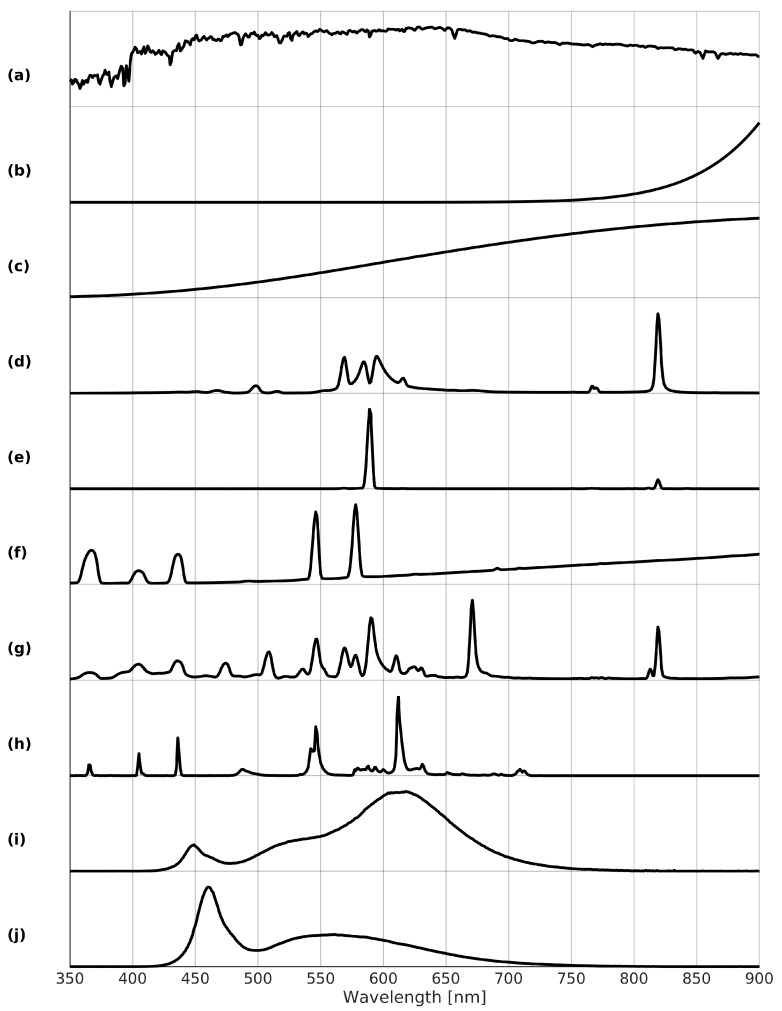
Typical normalized emission spectra by NOAA [17] and GUAIX [20] for different nighttime radiation sources (stacked offset for clarity): (**a**) cloud-free full moon; (**b**) fire, 700 K; (**c**) incandescent lamp; (**d**) high-pressure sodium lamp; (**e**) low-pressure sodium lamp; (**f**) mercury vapor lamp; (**g**) metal halide lamp; (**h**) fluorescent lamp; (**i**) warm-white LED lamp; (**j**) cold-white LED lamp.

The most prominent of those is the moon, reflecting sunlight arriving at its surface onto Earth. Hence, the moon is actually not a light source in itself, but instead acts as a reflecting object. The intensity of moonlight (0.1 lm/m${}^{2}$, cloud-free full moon) is rather small in comparison to sunlight (100,000 lm/m${}^{2}$, cloud-free full sun) or artificial lighting (10 lm/m${}^{2}$, lighted parking lot). In contrast to artificial lighting, however, the emitted light is not focused, but instead spatially homogeneous across the surface. Therefore, depending on the moon phase angle and with increasing elevation, moonlight becomes significant, even though its intensity is relatively limited. For example, moonlight is crucial in the detection of clouds from DMSP-OLS or NPP-VIIRS-DNB imagery, which is their principal focus and also explains the low detection limits of these sensors achieved also by the coarse spatial resolutions. Furthermore, moonlight facilitates the possibility to observe snow and ice features. Compared to the typical spectra of artificial lighting, moon spectral irradiances are relatively spectrally homogeneous across the VIS/NIR (Figure 2a). Most classifications and indices deal well with such offsets. For that reason and because of the relatively small illumination of moonlight especially for the considered fine spatial resolutions, namely the spectral radiances combine in particular one light source and one surface, the moon is not considered. Furthermore, the moon irradiance is modeled straightforwardly [19]. Approximating the surface reflectances using existing daytime imaging, e.g., Sentinel-2 or daytime acquisitions of the nighttime satellite, (or less adequate assuming a constant average value) and approximating the atmospheric compositions using existing operational services, e.g., ECMWF, (or less adequate assuming a constant standard atmosphere) allows estimating and eliminating this mixture.

Fires are natural (e.g., wildfires, volcanos) or artificial (e.g., fuel lamps, gas flares) radiation sources and considered in Section 2.2.

Further natural nighttime radiation sources, such as auroras, nightglow, lightning, and bioluminescence, either occur rarely or have insufficient intensities to be reasonably detected, here. For that reason, they are not considered.

### 2.2. Artificial Radiation Sources

The sources used by humans to produce lighting have changed drastically throughout history going from open fires to candles and oil lamps over natural gas to electrical light. For example, by [18] it was figured out that, among artificial light sources, HPS lamps were responsible for about half of the artificial light in the European Union in 2015, although a trend towards the use of LED lamps is to be expected. We give an overview of the most common exterior lighting types used and a description of their principal emission peaks, i.e., those wavelengths for which a particular lighting type emits most of its light.

Fire is a relatively common source of nighttime radiation either natural, e.g., forest and grass fires, lava, or artificial, e.g., candles, liquid, and pressurized gas- and petroleum-based fuel lamps. Fire emission spectra (Figure 2b) are described using Planck’s law for blackbodies, whereby other, here minor, effects on the spectra, e.g., kind of fuel, oxidation of fuel, amount of pressure, are not considered. For example, for liquid fuel emission peaks at 1350 nm are obtained, whereas for pressured fuel mantles typically contain rare Earth oxides absorbing infrared radiation to glow white in the visible. For typical fires, color temperatures range between 400 and 1100 K.

Incandescent (Inc.) lamps emit light by heating a tungsten filament inside a vacuum enclosed by a glass bulb. When electricity passes through the filament, it heats up, thereby producing a spectrum similar to that of a blackbody of the same temperature. However, for these bulbs most of the light with an emission peak between 900 and 1050 nm falls in the infrared part of the spectrum (Figure 2c).

The next five lighting types are gas discharge lamps, which generate radiation by sending electricity through an ionized gas, thereby releasing energy in the form of photons. Different gasses typically result in their own characteristic emission lines. The lines are broadened by hot vapor or high pressure due to physical broadening mechanisms.

High- and low-pressure sodium (HPS/LPS) lamps are a kind of gas discharge lamps which use sodium in an excited state. They typically emit a bright yellow-orange light. For HPS the strongest emission peak is at 819 nm (Figure 2d). Further broadened lines lie between 560 and 620 nm. LPS exhibits a distinct narrow line at 589 nm due to absence of line broadening and a weak emission peak at 819 nm (Figure 2e).

Mercury vapor (MV) lamps are another kind of gas discharge lamps, using mercury and providing a more blue-green color because of its peak emissions between 540 and 580 nm (Figure 2f). In contrast to other discharge lamps, it additionally resembles the curve of an incandescent lamp, with a peak at 1250 nm.

Metal halide (MH) lamps are similar to mercury vapor lamps, but with an additional mixture of metal halides added to the mercury. Metal halide lamps generally have strong emission peaks at 671 and 819 nm, with other peaks strongly depending on the composition of the halides (Figure 2g).

Fluorescent (Fluor.) lamps are gas discharge lamps at low-pressure using fluorescence to produce radiation. Like with mercury vapor lamps, they make use of mercury. However, the inner surface of the glass tube in which the gas resides contains a fluorescent coating of phosphors. This results beside smaller infrared emissions in two main emission peaks at 544 and 611 nm (Figure 2h).

Warm-white and cold-white light emitting diodes (wLED/cLED) lamps consist of one or more LED, which are semi-conductors releasing photons by radiative recombination when injecting an electrical current. Different kinds of semi-conductors are used to create a wide range of colors. Therefore, there are no specific emission peaks for LED, however, they are identifiable by relatively symmetrically shaped emission bands and a lack of infrared emissions. White LED generally have two primary peaks, one in the blue and another one in the green to red range (Figure 2i for warm-white and j for cold-white). If the correlated color temperature (CCT) is at most 4000 K, it is warm-white and otherwise, it is cold-white. As a result of their long lifespan and high efficiency, LEDs are becoming more and more the standard for both indoor and outdoor lighting.

For each of the eight lighting types, we consider the spectra of [17] (by NOAA (National Oceanic and Atmospheric Administration)) and [20] (by GUAIX (Universidad Complutense de Madrid, Group of Extragalactic Astrophysics and Astronomical Instrumentation)) interpolated to the range from 350–900 nm in steps of 1 nm as it is the range common to both libraries and comprises the VIS/NIR range of interest having a focus on outdoor street lighting. For fires, the spectra of blackbodies with temperatures of 400 K, 700 K, 900 K, and 1100 K are considered.

While it is possible to give an extensive overview of a number of artificial light emitting sources of radiation in the VIS/NIR part of the EM spectrum during nighttime, relative worldwide frequencies of lighting types are difficult to determine.

### 2.3. Luminance

To determine the intensity of lamps, the European standard (EN) for street lighting is used as reference [21]. As a result that it is more relevant to know how much reflected light is seen by the human eye than to know the radiant flux of a lamp, standard values for average street luminance are given instead. The required minimum luminance depends on the type of street and ranges between 0.3–2.0 cd m${}^{-2}$. Together with measured data a maximum luminance of 4.0 cd m${}^{-2}$ is considered, here. From these luminances the corresponding bottom-of-atmosphere (BOA) radiances are deduced. The BOA radiances have to equal the integral over all spectral radiances and they are computed based on combined lamp spectra and surface reflectances.

### 2.4. Surface

**Figure 3:**
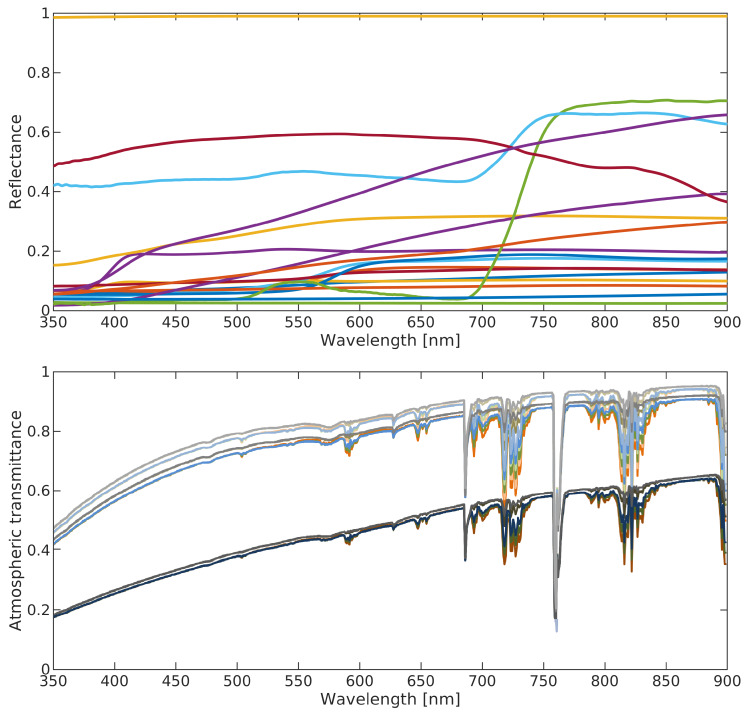
**Top**: Variation in surface reflectances for selected 18 spectra; **bottom**: Variation in atmospheric transmission curves for selected five atmospheric profiles and visibilities 10 km (minimum; dark lines), 55 km (mean), and 100 km (maximum; bright lines).

Surface reflectance data are required to generate reflected lamp emissions, namely indirect radiation towards the sensor. For this reason, 18 representative surface types including street asphalt, paved brick, road concrete, grass, snow, sand, wood, asphalt roof shingle, and a Spectralon with near-constant reflectance of 99%, which is considered as this is, here, similar to direct radiation towards the sensor, of [22] (by USGS (United States Geological Survey)) are used as source for surface reflectances (Figure 3, top).

### 2.5. Atmosphere

Radiative transfer is the physical process to transform BOA radiances to TOA radiances, by which radiation interacts with the atmospheric constituents. To quantify atmospheric impacts, the code by [23] is used. Since the focus is on cloud-free conditions, only such atmospheres are considered. How to differentiate between cloud-covered and cloud-free conditions even solely based on panchromatic images is illustrated by [24] especially for urban areas with high accuracy. Since the focus is on urban areas, urban aerosols and atmospheric profiles mid-latitude summer, mid-latitude winter, subarctic summer, subarctic winter, and tropical are included to cover a wide range of conditions. Together with visibilities between 10 and 100 km, that strongly determine transmittance, a range of 30–40% between highest and lowest atmospheric transmittance occurs (Figure 3, bottom). Without further assumptions, this also gives an indicator of the possible error range for atmospheric transmittance estimation, which is the largest error source in the estimation of radiant flux [25].

**Figure 4:**
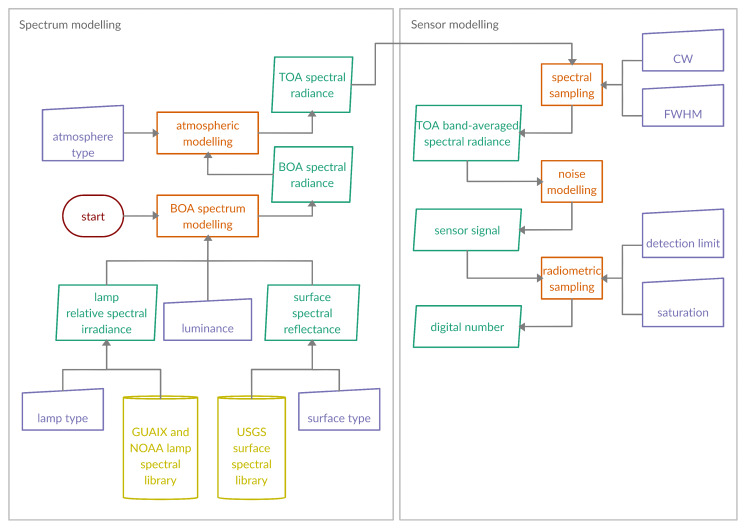
Framework for simulation of a spectral band’s sensor signal.

As illustrated in Figure 4, hence, a TOA radiance is generated for each of the eight considered lighting types by sampling uniformly at random among the corresponding lamp spectra of NOAA and GUAIX, luminances between 0.3 and 4.0 cd m${}^{-2}$ uniformly at random, as well as surface reflectances selected of USGS and for fire by sampling temperatures uniformly at random between 400 and 1200 K. Finally, one of five stated atmospheric profiles and between 10 and 100 km visibility selected uniformly at random are applied.

### 2.6. Satellite Sensor

The purpose of the satellite sensor is to produce radiance images on the focal plane array of an optical imaging system, where different spectral bands are sensitive to particular ranges of wavelengths. To compute for a band B the signal $L_{B}$ that arrives at a sensor through combining spectral radiances $L_{\lambda }$ of different wavelengths λ, the weighted average of the normalized effective radiance value over the detector bandpass $R_{B,\lambda }$ is considered, namely the band-averaged spectral radiance $L_{B}=\int_{0}^{\infty }L_{\lambda }R_{B,\lambda }d\lambda /\int_{0}^{\infty }R_{B,\lambda }d\lambda$ which is measured in W m${}^{-2}\;$sr${}^{-1}\;$nm${}^{-1}$. The detector bandpasses are difficult to synthesize accurately beforehand. As a general rule, however, such detector bandpasses are described by an analytical function which behaves as a combination of a rectangular function and a Gaussian function. Commonly, a symmetric super-Gaussian function $R_{B,\lambda }=2^{-{\left|2\left(\lambda -B_{\mathrm{CW}}\right)/B_{\mathrm{FWHM}}\right|}^{k}}$ is used, where $B_{\mathrm{CW}}$ and $B_{\mathrm{FWHM}}$ represent the center wavelength (CW) and full width at half maximum (FWHM) of band B and *k* denotes a parameter which defines the shape of the function. For high values of *k*, the function resembles a rectangular function, while for *k* close to 2 the Gaussian function is approximated. For optical remote sensing purposes, k=6 usually results in realistic detector bandpass functions [14]. Due to the robustness of optimization and due to production of detector bandpasses, we consider CW in steps of 1 nm and FWHM in steps of 5 nm.

The signal that constitutes the at-sensor radiance image does not only contain radiances originating from the already mentioned radiation sources. Additionally, it might include stray light in case the satellite is directly lit by sunlight and high energy particles. Moreover, noise can be introduced during the charge transfer process caused by detectors and electronic devices. Here, the focus lies on radiometric or optical imaging system noise because other noise sources, such as straylight, are relatively straightforward to model. To compare the amount of desired signal electron number $S_{B}$ to the level of noise electron number $N_{B}$, it is assumed that ${\mathrm{SNR}}_{B}=S_{B}/N_{B}$ as Signal-Noise-Ratio and ${\mathrm{NER}}_{B}=L_{B}/{\mathrm{SNR}}_{B}$ in W m${}^{-2}\;$sr${}^{-1}\;$nm${}^{-1}$ as Noise-Equivalent-Radiance. The largest radiometric noise contribution is a result of the random incidence of photons, thereby randomly generating photo-generated electrons, so-called photon shot noise. Assuming photon shot noise to be dominant and other contributions negligible, the noise electron number is rewritten as $N_{B}=\sqrt{S_{B}}$, thereby obeying a Poisson distribution. The signal electron number $S_{B}$ itself is retrieved by converting the incoming spectral radiance to electron content $S_{B}=L_{B}\cdot A\cdot \pi \cdot \tau \cdot \eta \cdot t\cdot B_{\mathrm{CW}}\cdot B_{\mathrm{FWHM}}$$/(\left(4{\left(f/\#\right)}^{2}+1\right)\cdot h\cdot c)$ with *A* being the detector’s effective area, τ is the system’s optical transmittance, η is the quantum efficiency, *t* is the effective integration time, f/# is the f-number, *h* is Planck’s constant, and *c* is the speed of light [26].

As a reference for the noise model, the recommendation by [13] on SNR is adopted, i.e., for a photopic spectral band P with $P_{\mathrm{CW}}=560\;$ nm and $P_{\mathrm{FWHM}}=100\;$ nm an SNR${}_{P}=10$ at a band-averaged spectral radiance $L_{P}=2.5\times 10^{-7}\;$W m${}^{-2}\;$sr${}^{-1}\;$nm${}^{-1}$. With the assumption of all other variables remaining identical, this results in ${\mathrm{NER}}_{B}=\sqrt{L_{B}P_{\mathrm{CW}}P_{\mathrm{FWHM}}/\left(L_{P}B_{\mathrm{CW}}B_{\mathrm{FWHM}}\right)}\cdot {\mathrm{NER}}_{P}$ and allows for a noise value to be taken from a Poisson distribution to be added to $L_{B}$.

Thus, as illustrated in Figure 4 for any given spectral band B, the TOA band-averaged spectral radiances $L_{B}$ are computed from the TOA radiance spectra and noise taken from a Poisson distribution with mean ${\mathrm{NER}}_{B}$ is added to the signal in order to end up with a realistic sensor signal. Note that the conversion to a digital number is not considered at this point; due to the fact that some sensor-specific qualities need to be known before detection limits and saturation values are determined.

### 2.7. Performance Metrics

For indices, we consider the mean absolute error (MAE) to measure errors, namely $\mathrm{MAE}=\sum_{1}^{n}\left|y_{i}-x_{i}\right|/n$, where $y_{i}$ is the estimated value and $x_{i}$ the true value.

For classifications, we consider the confusion matrix with true positive (TP), false positive (FP), false negative (FN), and true negative (TN). For example, the number of TP is defined by the number of correctly predicted positives, while the number of TN is defined by the number of incorrectly predicted negatives. With recall=TP/(TP+FN) describing the ability to correctly predict all positive instances and precision=TP/(TP+FP) describing the radio of correct predictions among those instances that have been predictive positive, it is F1=recall·precision/(recall+precision) considered as both measures are not reliable by themselves. For multiple classes the F1 is the mean over the F1 scores of all classes.

## 3. Results

As it is not effective to share full lamp spectral irradiances, technical descriptions of and management decisions on artificial lighting generally consists of only a limited number of performance parameters or indices. We investigate the most common spectral indices in lighting engineering, based on a report on road lighting and traffic signals of the European Commission [18,21]. These define how much light is emitted, how much of the emitted light is seen by the human eye, or how much light is emitted in the blue part of the EM spectrum. In addition to the discrimination of lighting types, also the perceived color of the emitted light is assessed.

For optimization the MAE concerning the lighting parameters and F1 score concerning the lighting classification are minimized or maximized based on the at-sensor radiances only. Namely information on the radiation source, luminance, surface, or atmosphere are not considered while estimating the values.

### 3.1. Luminous Efficacy of Radiation

In designing artificial lighting, achieving a high luminous efficacy is crucial. Luminous efficacy rates the amount of visible light that is produced, in lumen, divided by the total amount electrical power that is required, in watt. Therefore, it is a measure for the efficiency of a particular luminaire system. Not only does luminous efficacy take into account emissions outside of the visual spectrum, but also, for example, decreased lumen as a result of dirt collections on the luminaires or electrical power losses in control gears. As luminous efficacy is impossible to be estimated without any ground-based information, it is often interchanged with luminous efficacy of radiation (LER), which is computed as the ratio between luminous flux $\Phi_{v}=K_{\max}\int_{0}^{\infty }V\left(\lambda \right)\frac{d\Phi_{e}\left(\lambda \right)}{d\lambda }d\lambda$ with $K_{\max}$ being the greatest luminous efficacy which can theoretically be achieved at 555 nm, equaling 683 lmW${}^{-1}$, and V(λ) is the CIE photopic spectral luminous efficiency or the human eye’s relative sensitivity under well-lit conditions, and radiant flux $\Phi_{e}=\int_{0}^{\infty }\frac{d\Phi_{e}\left(\lambda \right)}{d\lambda }d\lambda$, namely $\mathrm{LER}=\Phi_{v}/\Phi_{e}$ [18].

Thus, a choice of two bands is physically required for the estimation of LER. A panchromatic band B0 is used to estimate the irradiance emitted across the full EM spectrum (i.e., denominator of equation) and a (near) luminance band B1 is used to estimate the amount of irradiation which is visible to the human eye (i.e., numerator of equation).

Band B0 estimates the amount of emitted irradiance, namely the radiant flux and is itself a light parameter playing one of the most important roles that corresponds to a certain luminaire system. The estimation of the radiant flux from band B0, however, is not straightforward, as it depends on a number of different parameters, e.g., surface reflection, atmospheric transmittance, and the ratio of emitted power within the measured spectrum. As in typical situations surface reflection and atmospheric transmittance are essentially constant and similar factors to bands B0 and B1, however, they basically cancel for LER. As band B0 covers the full EM spectrum, the signal is high and sensitive to all considered radiation types; it also serves as a panchromatic band and is used for normalization purposes. Whereas it is possible to estimate radiant flux of lamps, uncertainties remain relatively high. Usually, rather than the radiant flux, it is the required electrical power that is of interest. However, estimating the latter is further complicated by the need for data on electrical power efficacy, which describes the ability to transform electrical power into optical power.

Band B1 estimates the amount of visible light, namely the luminous flux and is also an important light parameter by itself, its detector bandpass function shall closely resemble the photopic spectral luminous efficiency curve V(λ). However, the form of a detector bandpass function differs from the form of V(λ). The optimal spectral band B1 to resemble V(λ) is reached for ${B1}_{\mathrm{CW}}=561\;$ nm and ${B1}_{\mathrm{FWHM}}=121\;$ nm, theoretically. Note that the CW differs slightly from the wavelength at which V(λ) reaches its maximum, i.e., 555 nm. This is ascribed to the asymmetrical form of V(λ) having a slightly positive skew. It is expected that this shift to higher wavelengths will be less significant in practice, as most lamps have almost no emissions around the larger base of the function, i.e., 650 nm.

The estimated LER, namely $\tilde{\mathrm{LER}}=a\cdot L_{B1}/L_{B0}$, is not only achieved by $L_{B1}/L_{B0}$ as these ratios do not yet represent LER values, but they need to be adjusted by applying a multiplication factor *a*. It is theoretically approximated by a $K_{max}\cdot {B0}_{\mathrm{FWHM}}/{B1}_{\mathrm{FWHM}}$. A practical approximation based on the ratios and LER using least-squares estimation is preferred.

In order to select the optimal parameters for B0 and B1, uniformly distributed sampling is used for the CW and FWHM. Based on the discussions, for B0, CW ranges between 500 and 750 nm and FWHM ranges between 300 and 550 nm, and for B1, CW ranges between 500 and 600 nm and FWHM ranges between 80 and 150 nm. Considering all possible combinations, the optimum is reached for the combination ${B0}_{\mathrm{CW}}=619\;$ nm and ${B0}_{\mathrm{FWHM}}=490\;$ nm (panchromatic) as well as ${B1}_{\mathrm{CW}}=556\;$ nm and ${B1}_{\mathrm{FWHM}}=125\;$ nm (green). Note that, as expected, B1 deviates slightly from those reached from approximating the photopic luminous efficiency function directly. An MAE for $\tilde{\mathrm{LER}}$ of 13 lmW${}^{-1}$ is reached. For comparison, the mean LER for the test data equals 307 lmW${}^{-1}$ with a standard deviation of 117 lmW${}^{-1}$. It is important to note that these LER are slightly higher than they will be in reality, since they are only based on the 350-900nm range, with data outside this range missing for the spectra. The theoretical estimation of *a* results in 174, while the practical estimation yields a=151. Applying these analyses to the band B1 recommended by [13], namely ${B1}_{\mathrm{CW}}=560\;$ nm and ${B1}_{\mathrm{FWHM}}=80\;$ nm, results in an MAE for $\tilde{\mathrm{LER}}$ of 46 lmW${}^{-1}$, indicating that the proposed bands offer a significant improvement for the estimation of the efficiency of artificial lighting.

Let us consider instead of the optimized the typical panchromatic band B0 with $B0_{\mathrm{CW}}=700\;$ nm and $B0_{\mathrm{FWHM}}=400\;$ nm. Here, the range 865–900 nm is covered, but it is relevant for fire and incandescent lamps only, where the radiant flux is only marginally emitted in VIS/NIR at all. However, using the optimized or typical panchromatic band, estimations on the radiant flux are derivable taking the lighting type into account. For all other lighting types there is almost no emission in this range. Here, the range 374–499 nm is not covered, but it is relevant for more than half of the considered lighting types, e.g., LED lamps produce strong emissions, however, HPS lamps do not.

### 3.2. Spectral G Index

Light pollution, especially in the blue part of the spectrum, has gained substantial attention. Examples are the disruptive effect of artificial lights on the nocturnal behavior of different species as well as on human health [27]. For a long time correlated color temperature has been the principal indicator for the amount of emitted blue light, despite its inability to sufficiently describe the spectrum of a lamp. However, the European Commission has published a report in which it recommends the use of the so-called spectral G index instead [18], which is computed as the total amount of luminous flux divided by the amount of radiant power emitted between 380 and 500 nm with high values corresponding to low blue light emissions, namely $G=2.5\log_{10}\int_{0}^{\infty }V\left(\lambda \right)\frac{d\Phi_{e}\left(\lambda \right)}{d\lambda }d\lambda /\int_{380}^{500}\frac{d\Phi_{e}\left(\lambda \right)}{d\lambda }d\lambda$. Note the similarities between the enumerator of this equation and the one of LER. Thus, a choice of two bands is physically required for the estimation of G, with a focus on the amount of blue light. However, the enumerator is estimated by the same spectral band B1. The denominator, on the other hand, comprises the sum of emissions between 380 and 500 nm, and therefore needs an additional band B2. This sum equals applying a rectangular detector bandpass with CW of 440 nm and FWHM of 120 nm. The optimal band for a super-Gaussian detector bandpass function will not, in practice, differ much from these values.

The estimated G, namely $\tilde{G}=2.5\cdot \log_{10}\left(a\cdot L_{B1}/L_{B2}\right)$, first ignores the logarithmic form, in order to more accurately estimate the multiplication factor *a*.

In order to select the optimal band B2, uniformly distributed sampling is used for the CW between 420 and 460 nm and FWHM between 100 and 140 nm based on the discussions. As expected, the optimal band B2 closely resembles the mentioned rectangular with ${B2}_{\mathrm{CW}}=443\;$ nm and ${B2}_{\mathrm{FWHM}}=120\;$ nm (blue). For a factor a=1.15 an MAE for $\tilde{G}$ of 0.081 is obtained. By comparing this result to the mean 1.875 and the standard deviation 1.923 of the data, adding a band in the blue part of the spectrum proves to be beneficial. The recommended bands by [13], occasionally criticized for its lack of a dedicated blue band, only reaches an MAE of $\tilde{G}$ of 0.569 with its scotopic band ${B2}_{\mathrm{CW}}=502\;$ nm and ${B2}_{\mathrm{FWHM}}=95\;$ nm. With common criteria suggesting G≥1.5 [18], an error of 0.081 is acceptable in most cases. Note that these accuracy values additionally depend on the characteristics of the sensor, i.e., detection limits, saturation, and the number of bits used for radiometric sampling. Hence, the error will be slightly larger in reality, but remains acceptable for a sensible choice of sensor parameters.

With most of modern streetlights being non-Planckian radiators more emphasis is placed on the estimation of the spectral G index, as opposed to estimating correlated color temperature.

### 3.3. Classification

In order to classify different radiation sources into their respective type, sensor data is compared to a spectral library with the means of a *k*-nearest-neighbor (KNN) classification. Put simply, a particular radiation source is labeled with the same class as the majority of its *k* nearest neighbors in feature space. KNN is used as it is robust. The goal is not to find the best possible classifier, but the classification method serves as a comparison measure to judge the usefulness of a particular band combination. When applying KNN, adding features might deteriorate the classification performance, even if it improves the classification of one of the classes. For example, if adding a particular band improves the classification of one lighting type, it might generate large distances for other lighting types, because of large variances in this band. To overcome these effects, with normalized bands considered as features, namely its signal is divided by the signal of the panchromatic band, KNN is applied to each possible combination of features individually. For each lighting type the best feature combination is determined by withholding the combination with the highest classification performance. To combine the resulting binary one-versus-all classification results, a weighted voting is performed, with the classification performance used as weights. In other words, if a particular lamp is classified as multiple types, the one with the best performing classifier will be the deciding. For the case where a lamp is not classified as any lighting type, it is labeled as no class.

Based on Figure 4, the spectral library includes two selected representative spectra for each of the eight considered lighting types combined with eight typical surface reflectances and normalized to luminance of 1 cd$\;m^{-2}$. Furthermore, four fires with temperatures equally distributed between 400 and 1100 K are included. Finally, all these spectral radiances are combined with the five stated atmospheric profiles at 20 and 75 km visibility each.

For each lighting type, KNN searches for the best possible combination of bands, including bands B1 and B2 that have been fixed. In order to select the optimal additional bands, uniformly distributed sampling is used for the CW between 350 and 900 nm and FWHM between 5 and 200 nm.

As a reference, the classification performance of the case is given, where no additional bands are added, i.e., by only making use of bands B0 for normalization, B1, and B2. Here, a mean F1 score of 0.620 is reached. The four bands suggested by [13] instead of B1 and B2, however, reached a mean F1 score of 0.791. Thus, it is evident that improvements are possible, and required, by including more bands. Table 1 gives an overview of the F1 scores for individual radiation source classes, for optimal band selections of 0, 1, and 2 additional bands. An examination of the optimal combination of 3 additional bands proves that improvements are minimal with a mean F1 score of 0.917 compared to 0.899 for 2 additional bands. One additional band results in a mean F1 score of 0.802. Hence, the addition of two bands is recommended in order to allow lighting type identification.

An optimum is reached for ${B3}_{\mathrm{CW}}=578\;$ nm and ${B3}_{\mathrm{FWHM}}=15\;$ nm (yellow-orange), in the case of one additional band B3. Corresponding mean F1 scores significantly increase to a similar mean F1 score than for the bands suggested by [13], but with one band less. For example, the largest improvement is seen for mercury vapor, which is clearly differentiated from other lighting types.

Although it is expected that the best combination of two additional bands includes a band identical, or at least similar, to mentioned band B3, this is not necessarily the case. The reason for this is that some parts of the spectra might possess high correlations with other parts of the spectra. In other words, joining the two best scoring spectral bands does not necessarily result in a better classification performance if they contain similar, correlated, information. The determination of two additional bands, therefore, needs to start from the situation with B0, B1, and B2 fixed, and with detector bandpasses for B3 and B4 being generated. In the case of two additional bands B3 and B4, an optimum is reached for ${B3}_{\mathrm{CW}}=576\;$ nm and ${B3}_{\mathrm{FWHM}}=15\;$ nm (yellow-orange) as well as ${B4}_{\mathrm{CW}}=815\;$ nm and ${B4}_{\mathrm{FWHM}}=35\;$ nm (near infrared). B3 does not, as is expected, differ much from the one optimal in the case of one additional band. The largest improvements are seen for HPS and LED classes as well as only two classes generate F1 scores lower than 0.8, i.e., fire and fluorescent.

A closer look at the confusion matrix in Table 2 reveals the reasons for these low values and details which types of misclassifications are to be expected. For example, fire is sometimes wrongly classified as an incandescent lamp or as a mercury vapor lamp. The confusion with mercury vapor lamps is probably solved by adding a narrow band around 545 nm, where mercury vapor lamps have one of their emission peaks. However, the relatively low occurrences of both classes in urban areas, and the relatively small improvement of introducing such a band aiming solely to distinguish between these two types, do not justify its consideration. Likewise, there is a high correlation between fluorescent and mercury vapor lamps. The reason for this lies in the manufacturing process of a fluorescent lamp. Similarly to mercury vapor lamps, fluorescent lamps make use of mercury gas, resulting in nearly identical emission spectra. Adding a narrow band around 610 nm probably solves this issue. However, again its consideration is not justified.

### 3.4. Correlated Color Temperature

In order to assess the perceived color of the light emitted by a particular lamp, its spectrum is compared to a range of blackbody radiators, which follow Planck’s law. The absolute temperature of the blackbody that most closely resembles the spectrum of the lamp, defines the so-called correlated color temperature (CCT) [18]. It needs to be noted that, while the computation of CCT values is relevant for lamps that closely resemble the spectrum of a Planckian source, e.g., in the case of incandescent lamps, it is no longer relevant for other lighting technologies such as gas discharge or LED lamps. Despite its limited ability to describe a lamp spectrum, CCT remains a widely applied indicator, as it is relatively straightforward to grasp its meaning.

Another frequently cited parameter to describe a light source spectrum is that of the color rendering index (CRI), which expresses a lamp’s ability to faithfully reproduce different colors along the spectrum, compared to a blackbody radiator with the same CCT. Typically, incandescent lamps have high CRI values close to the maximum value of 100. LPS lamps, on the other hand, have only one narrow peak in its spectrum and, therefore, yield low CRI values, near 0. As estimating CRI requires a very high spectral resolution its estimation is not considered, here.

Although it will most likely lose its value as a lighting metric, as it is limited in properly describing a lamp’s characteristics, CCT remains a valuable and frequently mentioned specification. For a proper estimation of CCT, a good distribution of spectral bands along the visible spectrum is required. Looking at the bands that are already fixed for LER, G, and classification of lighting types, B1 and B2 seem to be good candidates to cover the green and blue part of the spectrum. The part of the spectrum that is not covered by the existing bands is located in the red part of the spectrum. It is, therefore, expected that adding a single band in that wavelength range will significantly decrease the estimation error of CCT.

As expected, the optimum is reached for a band that covers the red part of the spectrum, namely ${B5}_{\mathrm{CW}}=610\;$ nm and ${B5}_{\mathrm{FWHM}}=75\;$ nm (red). By adding this band, MAE for the estimated CCT, namely $\tilde{\mathrm{CCT}}$ which is derived based on the estimated tristimulus values $\left(\tilde{X},\tilde{Y},\tilde{Z}\right)=\sum_{1\leq i\leq 5}\left(x_{i},y_{i},z_{i}\right)\times L_{Bi}/L_{B0}$ [28], is significantly improved from 994 K to 391 K. An additional advantage of including B5 is that it offers the possibility of generating true color imagery, with B2, B1, and B5 corresponding to the blue, green, and red band.

### 3.5. Performance Analyses

**Figure 5:**
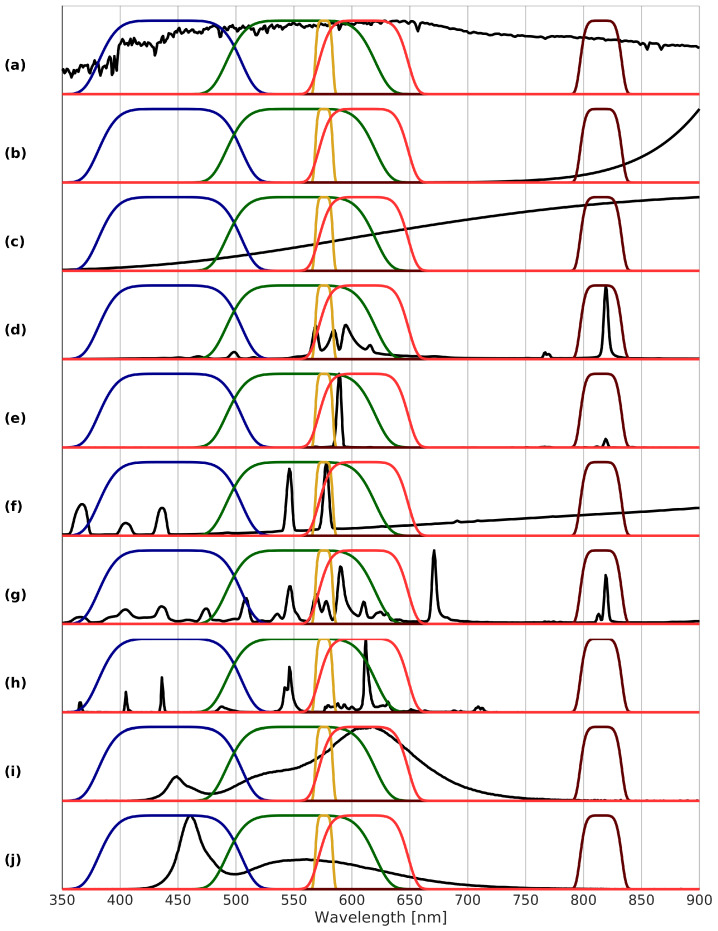
Selected bands and typical normalized emission spectra by NOAA [17] and GUAIX [20] for different nighttime radiation sources (stacked offset for clarity): Band 1 (green, 493–619 nm); Band 2 (blue, 383–503 nm); Band 3 (yellow-orange, 568–584 nm); Band 4 (near infrared, 797–833 nm); Band 5 (red, 572–648 nm). Note that Band 0 (panchromatic, 374–864 nm) is not plotted. (**a**) cloud-free full moon; (**b**) fire, 700 K; (**c**) incandescent lamp; (**d**) high-pressure sodium lamp; (**e**) low-pressure sodium lamp; (**f**) mercury vapor lamp; (**g**) metal halide lamp; (**h**) fluorescent lamp; (**i**) warm-white LED lamp; (**j**) cold-white LED lamp.

The recommended spectral bands are illustrated in combination with some typical lamp spectra in Figure 5 without the panchromatic band with 374–864 nm. What is immediately seen is the ability of bands B3 and B4 to distinguish between different lighting types. Additionally, there is a good spread of the different bands across the VIS/NIR spectrum, except for the wavelengths 650–800 nm. This unsurprisingly coincides exactly with that part of the spectrum where lamps typically emit no light. Due to the nature of nocturnal radiation sources, the choice of spectral bands differs significantly from the typical daytime optical sensors. This nighttime focus results in rather atypical bands, e.g., the narrow yellow-orange band B3 around 576 nm.

**Figure 6:**
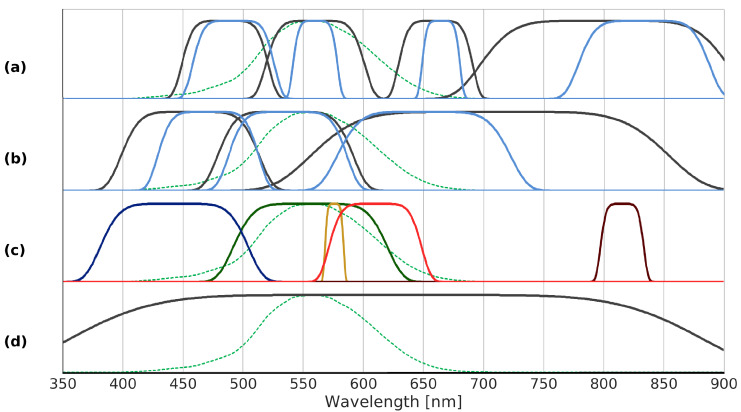
Dashed green line: CIE photopic spectral luminous efficiency V(λ); (**a**) black lines: Nightsat bands, blue lines: Sentinel-2 bands; (**b**) black lines: AC-5, blue lines: JL1-3B bands; (**c**) optimized bands B1–B5; (**d**) optimized panchromatic band B0.

A performance comparison for the selected bands with respect to other available band combinations, i.e., the proposal for Nightsat, 10 m bands of Sentinel-2, AC-5, and JL1-3B is given in Table 3, where the bands are illustrated in Figure 6. As the characteristics of the green band of JL1-3B and photographs taken by astronauts aboard the ISS are the same, the estimated LER are also the same. The blue bands are shifted by 10 nm only and therefore, the estimated spectral G indices are also similar. As the table reveals, a performance improvement was achieved for all relevant indices with the optimized bands. The Nightsat mission proposal, which is the standard reference with respect to nighttime VIS/NIR missions, does score relatively well in certain aspects. For example, the classification of radiation source types reaches similar results as the proposal with three multispectral bands. However, it does not succeed in estimating emissions in the blue part of the spectrum, as is reflected by the large MAE for the spectral G index.

### 3.6. Radiometric Resolution

As the conversion from sensor signal to a digital number as illustrated in Figure 4 is not considered, unlimited dynamic ranges and quantization are assumed. Detectors, however, have a detection limit, saturation, and bit depth. Assuming detectors have a linear response, the conversion for band B from an incoming band-averaged spectral radiance $L_{B}$ to ${\mathrm{DN}}_{B}$ is computed by ${\mathrm{DN}}_{B}=\left(L_{B}-{\mathrm{offset}}_{B}\right)/{\mathrm{gain}}_{B}$ with ${\mathrm{offset}}_{B}=L_{B,\min}$ and ${\mathrm{gain}}_{B}=\left(L_{B,\max}-L_{B,\min}\right)/{\mathrm{DN}}_{B,\max}$, where $L_{B,\min}$ and $L_{B,\max}$ represent the detection limit and the saturation of the sensor as well as ${\mathrm{DN}}_{B,\max}$ is the maximum digital number that can be attained, e.g., 255 for 8-bit images.

Recommendations for the detection limits and saturation of multispectral nighttime VIS/NIR sensors have been made by [13]. For a photopic spectral band with 510–610 nm detection limit and saturation of $2.5\times 10^{-7}$ and $2.5\times 10^{-1}\;$W m${}^{-2}\;$sr${}^{-1}\;$nm${}^{-1}$ are recommended. Here, band-averaged spectral radiances for the corresponding band B1 range between $6.3\times 10^{-8}$ and $4.4\times 10^{-5}\;$W m${}^{-2}\;$sr${}^{-1}\;$nm${}^{-1}$ for the lamps. Note that the lower limit considers land environments and challenges with the limited reflected light in aquatic environments are not covered. Especially the upper limit is significantly lower than the saturation recommended by [13], where the Luxor Sky Beam in Las Vegas, NV, USA, is used as reference. With a 42 billion candela tunnel of light, it is the strongest light beam in the world. Taking a linear response into account and ignoring less than 2% of all TOA radiances, for band B1 detection limit and saturation of $1\times 10^{-7}$ and $5\times 10^{-3}\;$W m${}^{-2}\;$sr${}^{-1}\;$nm${}^{-1}$ are recommended in the case of 16-bit as well as $1\times 10^{-7}$ and $3\times 10^{-4}\;$W m${}^{-2}\;$sr${}^{-1}\;$nm${}^{-1}$ in the case fewer bits are available. The detection limit recommended by [13], on the other hand, more or less conforms to the computed values, here. However, setting the detection limit to at most $5\times 10^{-8}\;$W m${}^{-2}\;$sr${}^{-1}\;$nm${}^{-1}$ extends the area of operation to the lighting of pedestrian and cycle zones.

For the other bands, the distribution of band-averaged spectral radiances follow that of band B1 for the lamps, with marginally lower values for the narrow band B3 and with the exception of two bands, i.e., the blue band B2 and the near infrared band B4. The rather low TOA radiances of band B2 between $1.4\times 10^{-10}$ and $2.7\times 10^{-5}\;$W m${}^{-2}\;$sr${}^{-1}\;$nm${}^{-1}$ is explained by the fact that some lamps, e.g., high- and low-pressure sodium lamps, barely emit blue light. Ignoring less than 12% of the smallest TOA radiances and with the importance of blue light emissions in mind, even for relatively low TOA radiances, it is recommended that the blue band has a slightly lower detection limit by a factor $10^{-1}$. Another difference is seen in band B4, where again some lamps, e.g., low-pressure sodium, fluorescent, and LED lamps, barely emit near infrared light, but also rather high TOA radiances of $1.8\times 10^{-3}\;$W m${}^{-2}\;$sr${}^{-1}\;$nm${}^{-1}$ are computed, belonging to some of the high-pressure sodium lamps. Therefore, by ignoring less than 33% of the smallest and 2% of the largest TOA radiances, it is recommended that this band has a higher dynamic range, with a saturation in the case of 16-bit at $5\times 10^{-3}$ and $8\times 10^{-4}\;$W m${}^{-2}\;$sr${}^{-1}\;$nm${}^{-1}$ in the case fewer bits are available. Increasing the saturation of band B4 is not only useful for high-pressure sodium lamps, but additionally increases the detection rate of fire.

Although the main focus is on urban areas, the detection of fire is an interesting by-product of a dedicated nighttime VIS/NIR sensor. Performing a similar analysis for fire spectra with temperatures between 400 and 1100 K illustrates that the highest TOA band-averaged radiances are not surprisingly located in band B4, in the near infrared part of the spectrum. However, even this band is not able to detect all fires, given the detection limit and saturation recommended for lamps. For a standard atmosphere a detection limit of $10^{-7}\;$W m${}^{-2}\;$sr${}^{-1}\;$nm${}^{-1}$ for B4 roughly corresponds to fires of 550 K, while a saturation at $10^{-3}\;$W m${}^{-2}\;$sr${}^{-1}\;$nm${}^{-1}$ for B4 roughly correspond to a temperatures of 750 K. However, the saturation is less of an issue, as TOA radiance values are lower in the panchromatic band B0, for example, thereby not exceeding the saturation threshold. As a consequence, most fires with temperatures exceeding 550 K are detectable by the recommended sensor. These temperatures cover most of the forest fires, meaning that the proposed spectral bands, with their detection limits and saturation, serve as an additional tool for fire detection programs. Although the VIS/NIR part of the spectrum does not cover the radiation peak of fires, as given by Wien’s displacement law, there is an important difference with respect to daytime optical sensors. The lower detection limits that are required for nighttime VIS/NIR sensors offer an opportunity to detect the lower TOA radiances that are emitted by fires in the VIS/NIR region, typically not visible to daytime VIS/NIR sensors.

For a standard atmosphere and a constant albedo of 20%, typical for road surfaces, TOA band-averaged spectral radiances for full moon conditions range between $1.5\times 10^{-7}$ for band B2 and $2.5\times 10^{-7}\;$W m${}^{-2}\;$sr${}^{-1}\;$nm${}^{-1}$ for band B5. As these values exceed the recommended detection limit, it is necessary to model out moonlight in most cases. With typical albedo values for snow and clouds around 95 and 70%, their respective TOA radiances range between $5\times 10^{-7}$ and $10^{-6}\;$W m${}^{-2}\;$sr${}^{-1}\;$nm${}^{-1}$. Thus, under full moon conditions, it is possible to detect both clouds and snow cover. As both effects are extended in size; a spatial binning results in detection capabilities even with reduced moonlight. However, in comparison to, e.g., NPP-VIIRS-DNB its ability to detect such phenomena is limited.

The computed performance metrics for LER, spectral G index, classification, and CCT are based on sensor signals before being converted into DN. This means that the results are slightly worse in a realistic setup, since certain small TOA radiance differences will be lost as a result of radiometric sampling. With the above-mentioned spectral bands and their recommended detection limits and saturation levels, additional analyses are carried out for different bit depths in Table 4.

For most bit depths, classification results are more or less stable, with the exception of an 8-bit conversion, which produces significantly deteriorated mean F1 scores. While the conversion to 10-bit still succeeds at classifying most of the radiation sources, the ability to estimate the luminous efficacy of radiation and spectral G index has drastically declined with respect to larger bit depths, with its values unacceptable for proper use. It is, therefore, recommended to apply a radiometric sampling of at least 12-bit, with higher bit depths not considerably better.

For each of the bands, typical TOA band-averaged spectral radiance values are calculated based on the selected lamp spectra, surface types, luminance recommendations, and atmospheric conditions. It is important to note here that some of these parameters are uniformly distributed between a minimum and a maximum value. Therefore, rather than covering a realistic distribution of values, it reflects a range of possibilities that is evenly distributed.

### 3.7. Spatial Resolution

The light emitted by artificial lighting sources does not only vary with wavelength, but also depends on the direction in which the light is emitted. The spatial resolution that is required for a VIS/NIR nighttime sensor depends completely on the objective of such a mission. It depends especially on the scale of the objects that need to be detected. For example, if the focus of a mission is on single-lamp level, a different spatial resolution is required, compared to city block level such as NPP-VIIRS-DNB. However, for such spatial resolutions, the multispectral approach makes little sense, since the signal that arrives at the sensor consists of a multitude of lamp signals and lamp types, turning the estimation of LER, spectral G index, and radiation type meaningless. Therefore, the focus will be on the single-lamp level here. It is sufficient to consider the panchromatic band only as reducing the spectral resolution as such is not likely to change the detectability of lamps.

**Figure 7:**
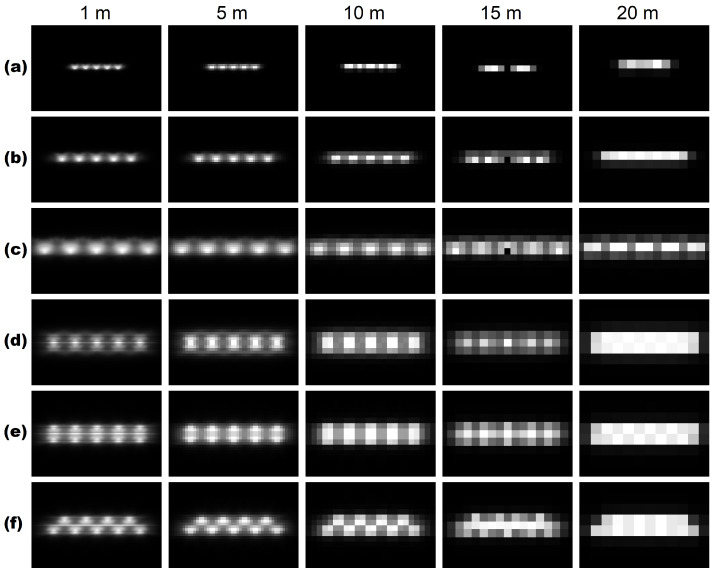
Single-row arrangement of five lamps with different spacing and mounting height at different spatial resolutions, with (**a**) spacing of 25 m, height of 6 m; (**b**) spacing of 40 m, height of 10 m; (**c**) spacing of 60 m, height of 18 m. Road lighting arrangements, spacing of 40 m, with (**d**) twin central arrangement; (**e**) two-sided opposite arrangement; (**f**) two-sided staggered arrangement of a dual carriageway.

To arrive at recommendations concerning the spatial resolution of a nighttime VIS/NIR sensor which focuses on artificial lighting, the spacing between different lamps, in combination with their mounting height, plays an important role. Typical values for these variables were derived from lighting engineering standards [21]. This lead to three different cases, i.e., a spacing of 25 m and mounting heights of 6 m for residential roads (Figure 7a); a spacing of 40 m and mounting heights of 10 m for roads with a mixed function (Figure 7b); and a spacing of 60 m and mounting heights of 18 m for major roads (Figure 7c). Lower spacing distances than 25 m do occur, but are not frequent. A typical luminous intensity distribution pattern is considered, which shows the intensity of emitted light for different directions.

According to the Nyquist sampling theorem, the sampling frequency shall be at least twice the highest frequency contained in a signal. Applying this logic here means that the required ground sampling distance should equal half of the spacing, or less, between neighboring lamps. With a minimum spacing of 25 m, this results in 12.5 m or less. Moreover, neighboring lamps usually possess similar characteristics, which makes the detection of individual lamps not necessarily required in all cases and a reduced ground sampling distance for multispectral bands than for the panchromatic band are feasible. For each of these cases, some sensible spatial resolution options are investigated in Figure 7. Notwithstanding the above-mentioned prediction, road lighting does not behave like a regular point source. Intensity distributions have a major influence on the positioning estimation of lamps. The results predicted by the Nyquist theorem, however, are confirmed by visual inspection that a spatial resolution of 10 m is feasible. As a target of lighting engineering is to create more homogeneous illumination patterns on the surface and the atmosphere blurs the shape, a spatial resolution less than 10 m is in this case required to distinguish lamps and between public and private lighting.

For larger-sized roads, such as dual-carriageways, not only the most common one-sided arrangement and single central arrangement occur that are sufficiently covered by the investigated single-row arrangement, but also a twin central arrangement (Figure 7d), a two-sided opposite arrangement (Figure 7e), and a two-sided staggered arrangement (Figure 7f) are common. Lamp spacings of 40 m are considered as a spacing of 25 m is not relevant, as it corresponds to relatively narrow roads in a residential area, for which a single-row arrangement is the preferred option.

A similar pattern as for single central arrangement with double the number of lamps is achieved for the twin central arrangement, as neighboring twin lamps are that close to each other that they are almost identical to single lamps. However, under certain conditions a thorough pattern analysis at a minimum spacing of at most half the distance between the centers of the light cones of the twin lamps allows the two lamps to be differentiated. Furthermore, as the light cones face each other, the task gets more complicated. For the two-sided opposite arrangement a similar situation occurs, the light cones are oppositely directed, which allows for a slightly coarser spatial resolution still enabling the discrimination of the lamps. Moreover, since the lamps exhibit identical characteristics, it is acceptable to classify them as a single lamp. It is considerably easier to detect single lamps for a two-sided staggered arrangement. Once more, as all lamps have a distance of more than 25 m to each other due to the carriage width, a comparable situation as for the single-row arrangement with a spacing of 25 m is present.

Given the recommended spatial resolution of 10 m and the relatively low detection limits, it is also possible to combine two panchromatic bands. The first band combines a high spatial resolution of 10 m with relaxed detection limits, thereby only focusing on lamp detection, while the second band combines lower detection limits with a relaxed spatial resolution, e.g., 20–25 m. This is also sufficient for the multispectral bands, for which a reduced spatial resolution of 40–50 m is still acceptable as lighting parameters typically do not change from lamp to lamp, but potentially from street to street. However, in this case a stronger mixture of the lamps spectra with spectra of residential and industrial lighting as well as vehicle lights have to be considered also in combination with skyglow [29].

## 4. Conclusions

With a focus on spectral characteristics, but also considering radiometric and spatial resolutions, the article performed and analyzed simulations to recommend performance parameters for nocturnal multispectral satellite imagery for urban areas as summarized in Table 5. The simulations accounted for all major contributions to the signal, namely typical theoretical fire spectra, lamp spectral libraries, standard luminance values for road surfaces, a surface reflectance library, estimations of atmospheric effects, and the sensor. Future research shall generate and consider fire spectral libraries in visible and near infrared (VIS/NIR) to thermal infrared to exploit the capabilities of enhanced nighttime satellite imagery for users of fire products. For urban areas the most important lighting parameters, namely luminous efficacy of radiation (LER) (with radiant flux and luminous flux), spectral G index (G), classification of lighting types (fire, incandescent lamps, high-pressure sodium lamps, low-pressure sodium lamps, mercury vapor lamps, metal halide lamps, fluorescent lamps, warm-white LED lamps, cold-white LED lamps) (Classif.), and correlated color temperature (CCT) are considered.

Reference radiances represent the mean of all considered TOA radiances and the corresponding Signal-Noise-Radio is derived according to Section 2.6, whereas all other parameters are already directly derived in Section 3.

The next step in this process of simulation is to generate more complex imagery based on measured lamps, measured reflectance spectra, digital surface models, cadastral maps, modeled cloud conditions and adding factors such as the moon, residential and industrial lighting as well as vehicle lights. Therefore, further ground, air-, and spaceborne measurements need to be integrated. Such more realistic data allow investigating how mixtures of different radiation sources and spatial resolutions influence, e.g., classification results and radiant flux estimations considering cloud parameters. Finally, the accuracies of the models and the parameters are to be covered more detailed which is of major importance for applications, too.

Nighttime images with high spectral and high spatial resolutions are a relatively unexplored field. The options for future research are, therefore, plentiful. One such area of interest is the estimation of radiant flux also considering inter- and intra-night changes of emissions, which enables recognition of changes in human activities, where research has already scratched the surface of this topic. Time-series of imagery at different scales are investigated to cover the dynamics of the urban lightscape [30]. Another such area of interest is the estimation of cutoff of lamps, i.e., non-cutoff, semi-cutoff, cutoff, or full-cutoff, to rate the amount of wasted light and glare which is a major lighting parameter not covered, here. Imagery acquired under different tilting angles are investigated.

With the mentioned areas of research only touching a part of the countless opportunities, simulating nocturnal imagery is a major step towards a deeper understanding of nighttime VIS/NIR remote sensing (missions) to reveal insights in the human activities shaping and changing the Earth. 

## Figures and Tables

**Table 1:** F1 scores per lighting type for 0, 1, and 2 additional bands.

	0 Additional Bands	1 Additional Bands	2 Additional Bands
Fire	0.537	0.741	0.791
Incandescent (Inc.)	0.744	0.785	0.856
High-pressure sodium (HPS)	0.697	0.838	0.981
Low-pressure sodium (LPS)	0.960	0.921	0.961
Mercury vapor (MV)	0.156	0.861	0.865
Metal halide (MH)	0.687	0.807	0.884
Fluorescent (Fluor.)	0.670	0.706	0.767
Warm-white LED (wLED)	0.554	0.729	0.926
Cold-white LED (cLED)	0.495	0.772	0.956
Mean	0.620	0.802	0.899

**Table 2:** Radiation source confusion matrix (relative). Columns and rows represent true classes and predicted classes.

	Fire	Inc.	HPS	LPS	MV	MH	Fluor.	wLED	cLED	Total
**Fire**	0.740	0.086	0.000	0.018	0.004	0.008	0.004	0.002	0.008	0.870
**Inc.**	0.104	0.846	0.000	0.000	0.016	0.000	0.000	0.000	0.000	0.976
**HPS**	0.000	0.000	0.984	0.000	0.010	0.012	0.000	0.000	0.000	1.006
**LPS**	0.000	0.000	0.000	0.928	0.000	0.000	0.004	0.000	0.000	0.932
**MV**	0.098	0.000	0.000	0.000	0.952	0.002	0.150	0.000	0.000	1.202
**MH**	0.016	0.000	0.014	0.006	0.002	0.894	0.060	0.012	0.018	1.022
**Fluor.**	0.000	0.000	0.000	0.000	0.004	0.038	0.730	0.082	0.050	0.904
**wLED**	0.000	0.000	0.000	0.000	0.000	0.000	0.048	0.904	0.000	0.952
**cLED**	0.000	0.000	0.000	0.000	0.000	0.008	0.002	0.000	0.924	0.934
**No class**	0.032	0.068	0.002	0.048	0.012	0.038	0.002	0.000	0.000	0.202

**Table 3:** Performance comparison with other band combinations. Note that results are based on a panchromatic band B0 with 374–864 nm. Overall accuracy (OA) is the total number of correctly classified instances divided by the total number of instances.

	Nightsat	Sentinel-2	AC-5	JL1-3B	B1–B3	B1–B4	B1–B5
Band 1 [nm]	450–520	459–525	400–512	430–512	383–503	383–503	383–503
Band 2 [nm]	520–600	542–578	480–590	489–585	493–619	493–619	493–619
Band 3 [nm]	630–690	649–680	560–850	580–720	568–584	568–584	568–584
Band 4 [nm]	700–900	780–886	-	-	-	797–833	797–833
Band 5 [nm]	-	-	-	-	-	-	572–648
LER [MAE]	46.07	114.40	46.58	68.73	12.66	12.66	12.66
G [MAE]	0.569	0.945	0.220	0.370	0.081	0.081	0.081
Classification [mean F1 score]	0.791	0.806	0.711	0.757	0.798	0.899	0.900
Classification [OA]	0.727	0.743	0.700	0.704	0.776	0.878	0.880
CCT [MAE]	1207	1134	1264	1442	1884	994	391

**Table 4:** Performance comparison for different bit depths, detection limit, and saturation considering B0-B5. ${}^{★}$ slightly different detection limit and saturation are considered. Overall accuracy (OA) is the total number of correctly classified instances divided by the total number of instances.

	*∞*-Bit	16-Bit ${}^{★}$	14-Bit ${}^{★}$	12-Bit ${}^{★}$	10-Bit ${}^{★}$	8-Bit ${}^{★}$
LER [MAE]	12.66	35.71	36.38	37.24	116.77	283.10
G [MAE]	0.081	0.118	0.128	0.169	0.715	1.804
Classification [mean F1 score]	0.900	0.842	0.861	0.865	0.839	0.690
Classification [OA]	0.880	0.794	0.774	0.787	0.768	0.662
CCT [MAE]	391	413	414	414	432	737

**Table 5:** Recommended performance parameters for nocturnal multispectral satellite imagery for urban areas. ${}^{★}$ normalization and single-lamp detection.

	B0	B1	B2	B3	B4	B5
spectral range [nm]	374–864	493–619	383–503	568–584	797–833	572–648
spectral range [color]	pan	green	blue	yellow-	near	red
				orange	infrared	
spatial resolution [m]	10	20	20	20	20	20
applications	${}^{★}$, LER	LER, G	G			
	Classif.	Classif.	Classif.	Classif.	Classif.
	CCT	CCT	CCT	CCT	CCT	CCT
bit depth [bits]	12	12	12	12	12	12
detection limit [W m${}^{-2}\;$sr${}^{-1}\;$ nm${}^{-1}$]	$1\times 10^{-7}$	$1\times 10^{-7}$	$1\times 10^{-8}$	$8\times 10^{-8}$	$2\times 10^{-7}$	$1\times 10^{-7}$
reference radiance [W m${}^{-2}\;$sr${}^{-1}\;$ nm${}^{-1}$]	$1\times 10^{-6}$	$3\times 10^{-6}$	$5\times 10^{-7}$	$3\times 10^{-6}$	$4\times 10^{-6}$	$4\times 10^{-6}$
saturation [W m${}^{-2}\;$sr${}^{-1}\;$ nm${}^{-1}$]	$3\times 10^{-4}$	$3\times 10^{-4}$	$3\times 10^{-5}$	$2\times 10^{-4}$	$8\times 10^{-4}$	$3\times 10^{-4}$
Signal-Noise-Ratio at reference radiance	24	23	6	14	15	37

## References

[1] Ghosh T., Hsu F.C. (2019). Advances in Remote Sensing with Nighttime Lights. Remote Sens..

[2] Levin N., Kyba C.C.M., Zhang Q., Sánchez de Miguel A., Román M.O., Li X., Portnov B.A., Molthan A.L., Jechow A., Miller S.D. (2020). Remote sensing of night lights: A review and an outlook for the future. Remote Sens. Environ..

[3] Croft T.A. (1978). Nighttime images of the Earth from space. Sci. Am..

[4] Bennett M.M., Smith L.C. (2017). Advances in using multitemporal night-time lights satellite imagery to detect, estimate, and monitor socioeconomic dynamics. Remote Sens. Environ..

[5] Miller S.D., Straka W., Mills S.P., Elvidge C.D., Lee T.F., Solbrig J., Walther A., Heidinger A.K., Weiss S.C. (2013). Illuminating the capabilities of the Suomi National Polar-orbiting Partnership (NPP) Visible Infrared Imaging Radiometer Suite (VIIRS) Day/Night Band. Remote Sens..

[6] de Miguel A.S., Kyba C.C.M., Aubé M., Zamorano J., Cardiel N., Tapia C., Bennie J., Gaston K.J. (2019). Colour remote sensing of the impact of artificial light at night (I): The potential of the International Space Station and other DSLR-based platforms. Remote Sens. Environ..

[7] Zhang G., Zhou Q., Yu B., Zhong X. (2019). The Design, Data Processing and Applications of Luojia 1-01 Satellite. Sensors.

[8] Levin N., Johansen K., Hacker J.M., Phinn S. (2014). A new source for high spatial resolution night time images: The EROS-B commercial satellite. Remote Sens. Environ..

[9] Pack D.W., Hardy B.S. CubeSat nighttime lights. Proceedings of the AIAA/USU Conference on Small Satellites.

[10] Zheng Q., Weng Q., Huang L., Wang K., Deng J., Jiang R., Ye Z., Gan M. (2018). A new source of multi-spectral high spatial resolution night-time light imagery: JL1-3B. Remote Sens. Environ..

[11] Levin N., Phinn S. (2016). Illuminating the capabilities of Landsat 8 for mapping night lights. Remote Sens. Environ..

[12] Kyba C., Küster T., Sánchez de Miguel A., Baugh K., Jechow A., Hölker F., Bennie J., Elvidge C.D., Gaston K.J., Guanter L. (2017). Artificially lit surface of Earth at night increasing in radiance and extent. Sci. Adv..

[13] Elvidge C.D., Cinzano P., Pettit D.R., Arvesen J., Sutton P., Small C., Nemani R., Longcore T., Rich C., Safran J. (2007). The Nightsat mission concept. Int. J. Remote. Sens..

[14] Segl K., Richter R., Küster T., Kaufmann H. (2012). End-to-end sensor simulation for spectral band selection and optimization with application to the Sentinel-2 mission. Appl. Opt..

[15] Kuechly H.U., Kyba C.C.M., Ruhtz T., Lindemann C., Wolter C., Fischer J., Hölker F. (2012). Aerial survey and spatial analysis of sources of light pollution in Berlin, Germany. Remote Sens. Environ..

[16] Kruse F.A., Elvidge C.D. Identifying and mapping night lights using imaging spectrometry. Proceedings of the IEEE Aerospace Conference.

[17] Elvidge C.D., Keith D.M., Tuttle B.T., Baugh K.E. (2010). Spectral identification of lighting type and character. Sensors.

[18] Donatello S., Quintero R.R., Caldas M.G., Wolf O., Van Tichelen P., Van Hoof V., Geerken T. (2019). Revision of the EU Green Public Procurement Criteria for Road Lighting and Traffic Signals.

[19] Kieffer H.H., Stone T.C. (2005). The spectral irradiance of the moon. Astron. J..

[20] Tapia C., de Miguel A.S., Zamorano J. (2015). LICA-UCM Lamps Spectral Database.

[21] Fotios S., Gibbons R. (2018). Road lighting research for drivers and pedestrians: The basis of luminance and illuminance recommendations. Light. Res. Technol..

[22] Kokaly R.F., Clark R.N., Swayze G.A., Livo K.E., Hoefen T.M., Pearson N.C., Wise R.A., Benzel W.M., Lowers H.A., Driscoll R.L. (2017). USGS Spectral Library Version 7: U.S. Geological Survey Data Services 1035.

[23] Mayer B., Kylling A. (2005). The libRadtran software package for radiative transfer calculations-description and examples of use. Atmos. Chem. Phys..

[24] Joachim L., Storch T. (2020). Cloud Detection for Night-Time Panchromatic Visible and Near-Infrared Satellite Imagery. Proc. ISPRS.

[25] Cao C., Bai Y. (2014). Quantitative analysis of VIIRS DNB nightlight point source for light power estimation and stability monitoring. Remote Sens..

[26] Eismann M.T. (2012). Hyperspectral Remote Sensing.

[27] Cho Y., Ryu S.H., Lee B.R., Kim K.H., Lee E., Choi J. (2015). Effects of artificial light at night on human health: A literature review of observational and experimental studies applied to exposure assessment. Chronobiol. Int..

[28] Wyszecki G., Stiles W.S. (1982). Color Science: Concepts and Methods, Quantitative Data and Formulae.

[29] Jechow A., Kyba C.C.M., Hölker F. (2020). Mapping the brightness and color of urban to rural skyglow with all-sky photometry. J. Quant. Spectrosc. Radiat. Transf..

[30] Dobler G., Ghandehari M., Koonin S.E., Nazari R., Patrinos A., Sharma M.S., Tafvizi A., Vo H.T., Wurtele J.S. (2015). Dynamics of the urban lightscape. Inf. Syst..

